# Unlocking the Luminescent Potential of Fish-Scale-Derived Carbon Nanoparticles for Multicolor Conversion

**DOI:** 10.3390/ijms252010929

**Published:** 2024-10-11

**Authors:** Najeeb S. Abdulla II, Marvin Jose F. Fernandez, Bakhytzhan Baptayev, Mannix P. Balanay

**Affiliations:** 1 Chemistry Department, Western Mindanao State University, Zamboanga City 7000, Philippines; najeeb.abdulla@wmsu.edu.ph; 2 Department of Chemistry, Mindanao State University-Iligan Institute of Technology, Iligan City 9200, Philippines; marvinjose.fernandez@g.msuiit.edu.ph; 3 National Laboratory Astana, Nazarbayev University, 53 Kabanbay Batyr Ave., Astana 010000, Kazakhstan; bbaptayev@nu.edu.kz; 4 Chemistry Department, Nazarbayev University, 53 Kabanbay Batyr Ave., Astana 010000, Kazakhstan

**Keywords:** response surface methodology, Box–Behnken design, hydrothermal synthesis, tamban, Sardinella, solid-state fluorescence, circular economy

## Abstract

This study introduces a novel approach to addressing environmental issues by developing fish-scale carbon nanoparticles (FSCNPs) with a wide range of colors from discarded fish scales. The process involves hydrothermally synthesizing raw tamban (Sardinella) fish scales sourced from Universal Canning, Inc. in Zamboanga City, Philippines. The optimization of the synthesis was achieved using the response surface methodology with a Box–Behnken design. The resulting FSCNPs exhibited unique structural and chemical properties akin to carbonized polymer dots, enhancing their versatility. The solid-state fluorescence of these nanoparticles can be modulated by varying their concentration in a polyvinylpyrrolidone matrix, yielding colors such as blue, green, yellow, and red-orange with Commission Internationale de l’Eclairage coordinates of (0.23, 0.38), (0.32, 0.43), (0.37, 0.43), and (0.46, 0.48), respectively. An analysis of the luminescence mechanism highlights cross-linking emissions, aggregation-induced emissions, and non-covalent interactions, which contribute to concentration-dependent fluorescence and tunable emission colors. These optical characteristics suggest that FSCNPs have significant potential for diverse applications, particularly in opto-electronic devices.

## 1. Introduction

Today’s environmental landscape is affected by the increasing challenge of waste management, which is a direct result of increasing population, industrial growth, and urbanization. This proliferation of waste threatens both our ecosystems and public health [1]. However, a new way of looking at waste management is emerging, one that sees waste as a resource and moves us towards a circular economy. This paradigm shift, in which waste is transformed into valuable materials and energy, is supported by scientific innovations that are driving the adoption of sustainable practices. In this circular economy, waste is no longer a burden, but a springboard for environmental protection and economic vitality. By harnessing the hidden potential of discarded materials, we can bring about positive change and ensure a healthier, more sustainable planet. Exploring the hidden value of waste, particularly in the field of advanced technologies, points the way to a future where waste equals resource regeneration. Our goal is to use sustainable materials and products not only to reduce environmental impact, but also to promote a robust, lasting ecosystem.

The idea of using value-added products and functional materials from waste materials is not new [2,3,4]. One promising solution is the reuse of waste products to produce sustainable and environmentally friendly chemicals that can be used as building blocks. These chemicals contain a variety of functional groups and can be used as starting materials for the production of high-quality advanced materials. Biomass, which is often discarded or underutilized, can be used as a low-cost feedstock for various applications. In fact, biomass-derived precursors can sometimes be even better than pure chemical precursors. For example, research has shown that biomass-derived carbon compounds perform better than synthetic carbon materials in various applications [5].

Fish scales are often regarded as a waste product of aquaculture. Every year, between 7.2 and 12 million tons of fish scales are thrown away worldwide [6]. However, fish scales have excellent mechanical properties and good biocompatibility, making them potentially useful in various fields such as engineering, electronics, and materials science [7]. The highly organized microstructures of fish scales are similar to the microstructures of hard human tissue [6]. Despite their potential, there is currently no commercial use of fish scales. This lack of value has led to environmental degradation and material waste. The reuse and conversion of fish scales could therefore reduce the environmental impact and create new opportunities for the commercial sector.

It is estimated that 19% of global electricity consumption is used for lighting [8]. With the phasing out of incandescent bulbs, energy-efficient and environmentally friendly lighting solutions are becoming increasingly popular. The most promising lighting technology is solid state lighting (SSL), which includes both light-emitting diodes (LEDs) and organic light-emitting diodes (OLEDs). LED efficiency has improved significantly in recent years, but it must meet higher energy performance standards in the net-zero scenario by 2030. Further progress could be made through the development of advanced LED modules, such as phosphors that can convert near-UV or blue radiation into visible radiation to produce white light. This field of research is growing rapidly, but the search for new phosphor materials with optimized emission efficiency, chemical and thermal stability, color conversion, and manufacturing costs is still crucial.

There are various studies that have investigated the use of fish scales in different areas. For example, carbon dots from bass scales have been used as photocatalysts [9], nanostructured particles from crucian carp scales for the detection of Fe^3+^ ions [10], nanoparticles from grass carp scales for the detection of glutathione [11] and carbon dots from perch and mullet scales for the production of UV-blocking films [12]. In addition, researchers in the field of optoelectronics have developed LEDs consisting of carbon nano-onions from fish scales that show promising fluorescence properties [13]. Our research aims to address a major limitation in the field of optoelectronics, namely the restriction of the luminescence of certain materials to bright blue. This limitation poses a challenge in achieving multicolored light emissions. To overcome this limitation, we have explored an innovative and sustainable approach using fish scales as precursors for the synthesis of carbon nanoparticles, which are then used to produce phosphors. Using a straightforward and environmentally friendly synthesis technique, we were able to modulate the properties of these phosphors and give them the remarkable ability to convert light into different colors. This development offers a promising solution to achieve multicolored light emission, which is an important requirement for many optoelectronic applications, including those in the Internet of Things (IoT) domain, where diverse and adaptable light sources are crucial for sensor and communication technologies. Our research shows that it is possible to use sustainable and unconventional but abundant waste materials, such as fish scales, as feedstock for the development of high-performance phosphors. This breakthrough opens up new possibilities for the development and manufacturing of advanced optoelectronic devices and provides a sustainable and environmentally friendly solution for the industry. By utilizing these abundant waste materials, we can reduce the environmental impact of conventional manufacturing methods while improving the performance of optoelectronic devices. This innovative approach has the potential to revolutionize the industry and pave the way for more sustainable and efficient technologies.

## 2. Results and Discussion

### 2.1. Optimization of Synthesis Parameters Employing the Box–Behnken Design and Response Surface Methodology Analysis

The FSCNPs were synthesized by the conventional hydrothermal digestion method using raw tamban fish scales as the precursor material. To effectively refine the synthesis conditions, response surface methodology was applied using a Box–Behnken design (BBD), a mathematical and statistical technique that aims to uncover empirical relationships between the desired outcomes and the influential independent variables [14]. RSM is characterized by its efficiency in refining complex reaction parameters and plays a central role in optimization efforts in the field of chemistry. By minimizing the number of experiments required, it provides comprehensive insight into the intricate relationships between variables and reactions, ultimately improving efficiency, resource utilization and product quality. The choice of the Box–Behnken experimental design is based on its balanced experimental framework, which requires fewer runs compared to other experimental designs while still providing reliable results. This efficiency proves crucial in complex or resource-intensive experiments and can be easily adapted to different research contexts. In our experiments, the key variables—reaction temperature, reaction time, and the amount of deionized water—were systematically investigated within specific ranges to evaluate their effects on fluorescence intensity, guided by the existing literature [15,16,17]. Table S1 shows the experimental BBD factors and level design for optimizing the preparation conditions of FSCNPs, and Table S2 shows the corresponding results of 15 runs. From the results, the largest AUC can be observed at the reaction parameters (180 °C, 7 h, 5 mL). Figure S1 shows a graphical representation of the highest AUC compared to the other reaction parameters in the experimental range. Minitab software version 21 was used to derive a regression equation for the reaction surface model (Equation (1)), which illustrates how different factors influence the reaction variable.

(1) $$Y^{\prime }=15.62+0.961X_{3}-0.1531X_{1}-0.315X_{2}-0.04695X_{3}^{2}+0.000567X_{1}^{2}\;-0.0555X_{2}^{2}-0.003801X_{3}X_{1}+0.01249X_{3}X_{2}+0.00708X_{1}X_{2}$$

where *X*_1_ is the reaction temperature, *X*_2_ is the reaction time, and *X*_3_ is the volume of deionized water. However, terms with *p*-values > 0.05 were classified as insignificant (Table S3), which led to a refinement of the equation (Equation (2)).

(2) $$Y^{\prime }=15.62+0.961X_{3}-0.1531X_{1}-0.315X_{2}-0.04695X_{3}^{2}\;+0.000567X_{1}^{2}-0.0555X_{2}^{2}-0.003801X_{3}X_{1}+0.00708X_{1}X_{2}$$

The equation highlights how various factors such as the sample amount, temperature, and time, as well as their interactions, influence the fluorescence intensity (response variable). Interestingly, increasing DI water (*X*_3_) has a statistically significant positive effect, suggesting a higher DI water amount generally leads to a stronger response. In contrast, both temperature (*X*_1_) and time (*X*_2_) show statistically significant negative effects, indicating a tendency for the response to decrease with increases in either factor. Furthermore, the contour plots illustrated in Figure S2 provide additional support for the proposed equation. In the case of DI water, the top row (row a) shows a shift of the highest fluorescence intensity (FI AUC) region (darker green shading) towards higher temperatures as the DI water volume increases. This suggests a positive correlation between DI water amount and FI AUC at a given temperature. This trend is also evident in the bottom row (row c), where FI AUC increases from left to right across each plot with increasing DI water volume, regardless of the specific heating temperature. This corroborates the finding that higher DI water volume enhances fluorescence intensity. Conversely, the middle (row b) and bottom (row c) contour plots, where DI water volume is constant, reveal a negative correlation between heating temperature and FI AUC. As the temperature increases from left to right across each plot, the FI AUC generally decreases. Additionally, the overall trend across all plots shows a decrease in FI AUC as the synthesis conditions shift towards higher temperatures (rightward) and longer heating times (downward). This further reinforces the observation that both temperature and time negatively impact fluorescence intensity.

Further analysis of the regression model reveals that the impact of temperature and experiment duration on fluorescence intensity is more complex than initially anticipated. While a positive coefficient for temperature ($X_{1}^{2}$) suggests an initial boost in response as temperature increases, this effect might plateau or even reverse at higher temperatures, indicating a potential point of diminishing returns. Similarly, the negative coefficients for sample amount ($X_{3}^{2}$) and time ($X_{2}^{2}$) suggest a concave curvature. This means that increasing either factor initially leads to a positive response in fluorescence intensity, but this effect weakens or even reverses at higher values. There might be an optimal range for both sample amount and time, beyond which further increases become detrimental to the desired outcome. While the analysis reveals complex interactions between these factors, the effect of some interactions might be less pronounced. The term representing the interaction between sample amount and time (*X*_3×2_) has a *p*-value of 0.208, which suggests it might not be statistically significant. This implies that the effect of increasing sample amount on fluorescence intensity might not be strongly dependent on the duration of the experiment (*X*_2_). However, the negative coefficient of the sample amount and temperature interaction term (*X*_3_*X*_1_) remains significant. This suggests an antagonistic effect, meaning the positive effect of increasing the sample amount weakens at higher temperatures. Conversely, the positive coefficient of the temperature and time interaction term (*X*_1_*X*_2_) indicates that the influence of temperature on fluorescence becomes more pronounced over time. There might be a specific time window where temperature has a more significant impact on the underlying process.

The ANOVA (Analysis of Variance) provided in Table S3 summarizes the results of the regression analysis using the quadratic model. This analysis assesses the significance of the different factors and their interactions in influencing the fluorescence intensity related to the synthesis of FSCNPs. The ANOVA reveals a highly significant response surface model that effectively explains 97.20% of the variation in the synthesis of FSCNPs. The fact that the blocking factor is not significant indicates that it did not introduce any systematic bias or variability into the experiment. This allows to focus on the main effects of the individual factors/parameters and their interactions, in understanding and optimizing the synthesis process. The individual linear effects of DI water, temperature, and time are all crucial, with positive contributions to the response variable. Additionally, the quadratic terms are also significant, indicating that the relationships between these factors and the response are not simply linear but also involve curvature. Importantly, the interaction between sample amount and temperature is significant, suggesting their effects on the response are interdependent. However, the interaction between sample amount and time is not significant. Notably, the interaction between temperature and time is highly significant, implying that the influence of temperature on the synthesis process depends on the reaction duration. The lack-of-fit observed is not statistically significant, indicating that the model’s predictions are generally reliable. Thus, the model underscores the importance of all three factors and their interactions in optimizing the synthesis of FSCNPs.

It is also worth identifying the most important factor that has the most significant effect on the FSCNPs; based on Figure S2, the contour plots favorably show that temperature appears to have a substantial influence on the synthesis of the FSCNPs. In most of the contour plots illustrated, the gradient of color change, representing changes in fluorescence intensity, is steepest along the temperature axis (horizontal axis). This indicates that even small changes in temperature lead to substantial differences in fluorescence intensity, suggesting a major impact on FSCNP synthesis. The strong influence of temperature is consistent across all three levels of DI water (row a). This suggests that temperature’s effect is not dependent on the specific amount of DI water used. The contour plots also show that the effect of temperature interacts with heating time. In some cases, the negative effect of temperature seems to be more pronounced at longer heating times. This further highlights the importance of temperature in the synthesis process, which is consistent with the work of Barati and Dela Cruz [15,17]. Investigating further, the relative impact of DI water and time on the synthesis of FSCNPs seems less pronounced than that of temperature. It appears that DI water (*X*_3_) has a slightly more pronounced effect on the synthesis of FSCNPs than time (*X*_2_). While DI water does influence the synthesis process, its impact is less critical compared to temperature. This suggests that a wider range within the region of DI water volumes can be successfully employed, as long as the chosen volume is sufficient to facilitate the reaction.

Thus, optimal conditions for the synthesis of the FSCNPs can also be identified using the RSM BBD model. Upon theoretical analysis, it was shown that the optimal parameters to use are the following: temperature = 180 °C, DI water amount = 3.9 mL, and time = 7 h. Table S4 shows the nine (9) optimization algorithm solutions computed, in which the stated optimal conditions are indicated.

The synthesis of FSCNPs is influenced by several parameters, among which temperature emerges as the most critical based on optimization results. While the volume of DI water also plays a role, it offers flexibility within a reasonable range that supports the reaction. Notably, lower temperatures (specifically in solutions 5–8) lead to a marked decrease in fluorescence intensity, underscoring temperature’s significant influence. In contrast, the impact of reaction time is less pronounced, though slightly shorter durations may provide a minor advantage. Given these findings, the optimal parameters for the further analysis and characterization of FSCNPs are identified as 180 °C heating for 7 h with 5 mL of DI water. RSM analysis defines the “optimal region” for DI water volume as approximately 3.86 mL to 5 mL, suggesting that minor deviations within this range do not significantly affect process performance. Selecting 5 mL ensures greater reproducibility and helps mitigate potential measurement inaccuracies inherent in the experimental setup. Preliminary validation experiments confirm that using 5 mL consistently yields desired outcomes, aligning with the robustness objectives of the study. This choice balances model predictions with practical considerations and prioritizes temperature, the most influential factor, to ensure the production of FSCNPs with high fluorescence intensity.

### 2.2. Characterization of FSCNP

**Figure 1:**
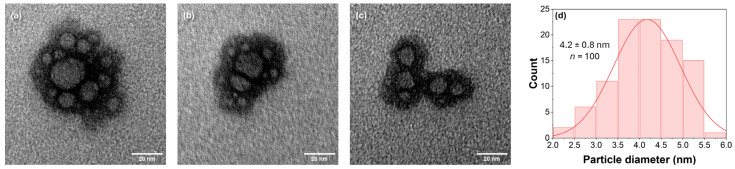
(**a**–**c**) TEM images of FSCNPs and (**d**) particle size distribution statistics of the individual carbon dots.

Transmission electron microscopy was used to analyze the size and morphology of carbon nanoparticles extracted from fish scales, which are crucial for influencing optical properties such as absorption and emission wavelengths. Figure 1 shows the clusters of nanoparticles observed by TEM, which have an average cluster size of 24.8 ± 6.7 nm. The aggregation of FSCNPs is attributed to the polymerization induced by the precursor material, which is characterized by distinct cross-linking points in Figure 1a–c. These wrappings of the amorphous polymer chains of the carbonized cores were previously observed by Lui and colleagues when they reacted trimethylolpropane tri (cyclic carbonate) ether with melamine, as a nitrogen source, to form the carbonized polymer dot [18]. Energy dispersive X-ray spectroscopy identified the elemental composition and showed C, N and O in an atomic ratio of 1.1:1.0:4.0, although the contribution of oxygen may be diluted due to analysis on a fluorine-doped SnO_2_ substrate (Figure S3). It should be noted that nitrogen has almost the same concentration as carbon, resulting in a high concentration of nitrogen in the matrix of carbon dots. This suggests that the fish-scale precursors are an effective source of self-doping. This self-doping approach allows for the easy incorporation of nitrogen into the carbon dot matrix through cross-linking, carbonization and/or polymerization, leading to an improvement in fluorescence properties and photoluminescence quantum yield by utilizing the ability of nitrogen to inject electrons into the carbon sites [19]. The TEM images show that the FSCNPs have an average diameter of 4.2 nm with a relative standard deviation (RSD) of the particle size distribution of 18% (*n* = 100) (Figure 1d), which falls within the range of the RSD of the particle size distribution of the synthesized high-quality carbon dots, which is between 10% and 37% [20].

**Figure 2:**
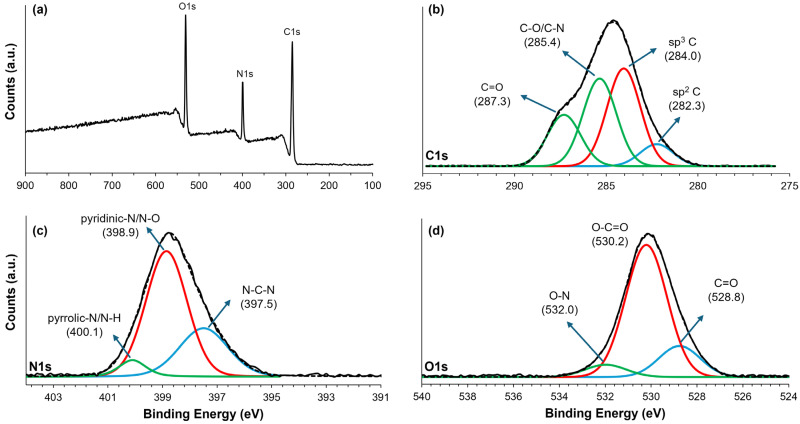
(**a**) XPS survey scan and high resolution XPS spectra of FSCNPs for (**b**) C1s, (**c**) N1s, and (**d**) O1s.

XPS analysis was used to evaluate the chemical composition and elemental states of the FSCNPs, as shown in Figure 2. The XPS full-scan spectra (Figure 2a) show the presence of carbon, nitrogen, and oxygen in the FSCNPs. Remarkably, the analysis identifies significant nitrogen content on the surface of the nanoparticles, which accounts for 20%, in comparison to the carbon content, which accounts for 41%. This finding confirms the higher concentration of nitrogen in FSCNPs. It is important to note that the differences in C/N ratio between the EDX and XPS results are due to the different methods of sample analysis. While EDX measures the composition of the bulk of the sample, XPS focuses on the composition of the surface. Any discrepancy between the composition of the surface and the composition of the bulk can lead to deviations in the measured ratios. In addition to the substantial nitrogen doping, the XPS spectra reveal the presence of abundant oxygen functional groups, which accounts for 39%. High-resolution XPS analysis (Figure 2b) has identified four distinct components in the C1s spectrum, revealing a diverse array of carbon species and functional groups. The peak at 282.25 eV signifies sp^2^ hybridized carbon, commonly found in aromatic or unsaturated structures, likely contributing significantly to the overall carbon network in the dots. At 284.05 eV, the peak corresponds to graphitic or aliphatic carbon (sp^3^ hybridized), indicating more saturated carbon structures that enhance the dots’ stability. Furthermore, the peak at 285.36 eV suggests the presence of C–O and potentially C–N bonds, indicating nitrogen-containing functional groups. Lastly, the peak at 287.31 eV indicates the presence of more oxidized carbon species, such as carbonyl or carboxyl groups, which may interact with nitrogen groups, particularly in the presence of amides or carboxylic acids [18]. The N1s XPS spectrum presented in Figure 2c shows three distinct peaks indicating a complex nitrogen environment in the material. The peak at 397.5 eV is associated with N-C-N species, indicating a nitrogen network integrated into a carbon framework that could improve the stability and electronic properties of the structure. The second peak at 398.9 eV corresponds to pyridinic- N and/or N-O species, indicating the presence of nitrogen in aromatic systems capable of donating electron density. The third peak at 400.1 eV likely relates to pyrrolic- N and/or N-H groups, indicating a different nitrogen bonding environment that may be involved in various reactions and serve as potential sites for hydrogen bonding, increasing the versatility of the material [21,22]. As for the O1s XPS spectra in Figure 2d, three peaks provide information about the oxygen functionalities present in the material. The peak at 528.8 eV, which is due to C=O groups, indicates the existence of carbonyl functions, which are important due to their electrophilic nature. The peak at 530.2 eV corresponds to the O-C=O groups, which indicate carboxylic acids or esters that can improve solubility and compatibility with other materials through strong hydrogen bonds. Finally, the peak at 532.0 eV is likely related to C-OH and/or O-N species, with hydroxyl groups improving hydrophilicity and surface properties. The presence of O-N species suggests interactions with the identified nitrogen functionalities and highlights a complex interplay that could further influence the stability and reactivity of the material [23].

**Figure 3:**
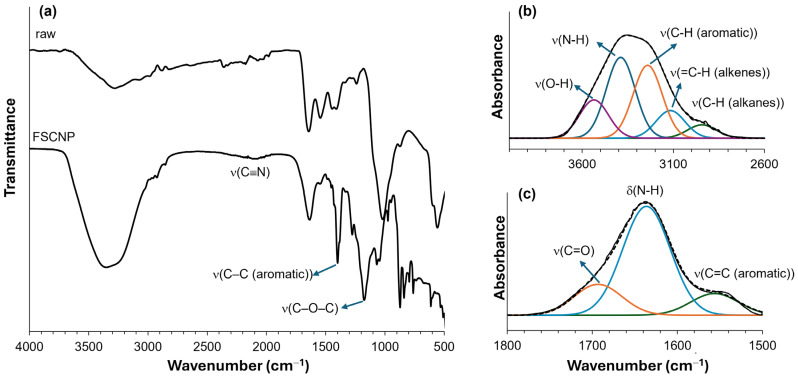
(**a**) FTIR spectra of the FSCNP and raw fish scales. Deconvolution of FTIR spectra in (**b**) 4000–2600 cm^−1^ and (**c**) 1800–1500 cm^−1^ regions.

The FTIR analysis was conducted to further identify the chemical species present in FSCNPs, as illustrated in Figure 3. Spectral analysis was performed both before and after the synthesis of FSCNPs (Figure 3a). After synthesis, an increased intensity was observed in the region between 2600 and 3600 cm^−1^. Deconvolution analysis suggested that this increase could be due to a combination of multiple functional groups, as indicated by the XPS spectra (Figure 3b). The peak at 3535 cm^−1^ is likely attributed to the -OH stretching vibration of the -COOH group, while the peak at 3389 cm^−1^ may represent N-H stretching vibration. Peaks at 3241, 3116, and 2943 cm^−1^ are associated with C-H stretching vibrations in the aromatic, alkene, and alkane species, respectively. The peak at 2137 cm^−1^ corresponds to C≡N. Further deconvolution analysis in the 1500 to 1800 cm^−1^ range helped in assigning the functional groups responsible for these peaks (Figure 3c). The peak at 1693 cm^−1^ is attributed to C=O stretching, 1636 cm^−1^ to N-H bending, and 1556 cm^−1^ to aromatic C=C stretching. The peak at 1400 cm^−1^ is assigned to C-C stretching in aromatic or conjugated bonds. The peak at 1175 cm^−1^ is likely due to C-O-C stretching, suggesting the presence of sp^2^ hybridized carbon atoms along with hydroxyl, carbonyl, and carboxylic functional groups. The Fourier Transform Infrared spectroscopy analysis showed that the material contains substantial amounts of amino, carboxyl, and other hydrophilic groups. These groups increase its solubility in water. Additionally, the diverse functional groups present on the surface of the FSCNPs provide significant versatility, enabling the customization of various properties, including tunability, solubility, biocompatibility, and stability.

**Figure 4:**
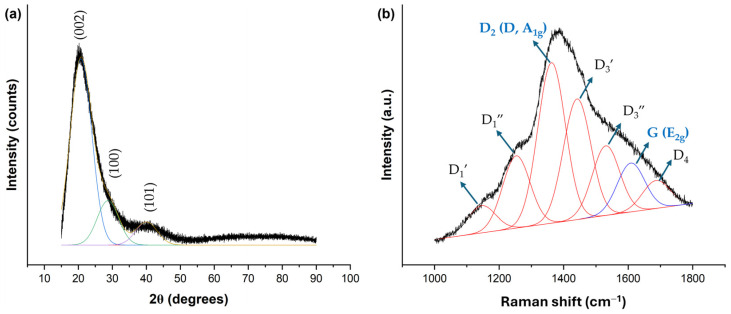
(**a**) XRD and (**b**) Raman spectra of FSCNPs. The spectra also include the deconvoluted peaks for further identification.

The X-ray diffractometry analysis of the FSCNPs structure, depicted in Figure 4a, reveals an asymmetric (002) peak at 20.78°, indicating the presence of additional bands. The deconvolution of the data identifies a peak at 28.71°, which may correspond to the (100) plane, suggesting the existence of sp^2^ hybridized carbon structures. Another peak at 40.04° could be associated with the (101) plane, indicating more complex short-range ordering that likely involves sp^3^ hybridized carbon atoms [24]. The (002) plane suggests some degree of graphitic ordering, and the interlayer distance (d) calculated from this peak is 0.43 nm. According to Bragg’s law, this distance indicates weak aromatic-layer attraction between the carbonaceous planes, as it is larger than the interlayer distance of graphite (d = 0.34 nm). The presence of -O and -N groups, identified through FTIR and XPS analyses, likely contributes to this effect. These findings suggest that the synthesis of fish scales primarily resulted in the formation of amorphous carbon structures rather than graphitization. In addition to the analyses performed using TEM, FTIR, XPS, and XRD, we also employed Raman spectroscopy to further enhance our findings. As shown in Figure 4b, we applied a 7-peak model to fit the Raman spectra in the one-photon scattering range of 1000–1800 cm^−1^. This approach allowed us to identify amorphous carbon in nanostructured carbon materials, achieving a fitting R^2^ value of 0.9979 [25]. The D_1′_ mode, observed at 1146 cm^−1^, corresponds to a combination of vibrational modes associated with chain stretching, including contributions from vinyl groups, C-H wagging motions, and heteroatoms as well as sp^2^ carbon atoms in defects and amorphous regions [26]. The peak at 1253 cm^−1^, identified as the D_1_″ mode, is likely related to various types of defects in carbon, such as point defects, edge planes, stacking faults, curved planes, or twisted planes [27]. The D_2_ mode, or simply the D mode, found at 1362 cm^−1^, is linked to vibrations with A_1g_ symmetry, resulting from disruptions in the graphitic lattice. These disruptions can be due to an increased number of edge planes relative to basal planes, reduced crystallite size, grain boundaries, amorphous carbon, and doping [26]. The D_3_′ and D_3_″ modes, characteristic of amorphous carbon, are represented by the peaks at 1442 and 1531 cm^−1^, respectively [27]. The peak at 1608 cm^−1^ corresponds to the G mode, which is attributed to in-plane vibrations with E_2g_ symmetry involving bond-stretching motions of sp^2^ carbon atom pairs [26]. Finally, the D_4_ mode, observed at 1687 cm^−1^, arises from the vibrations of graphene layers on surfaces resembling graphite [27]. The Raman spectroscopy analysis provides valuable insight into the structural characteristics of the amorphous carbon, particularly through the intensity of the D and G peaks. The ID/IG ratio of 3.26 indicates a higher degree of disorder or defects in the carbon material, a finding that is consistent with the XRD results.

### 2.3. Optical Properties of FSCNPs

**Figure 5:**
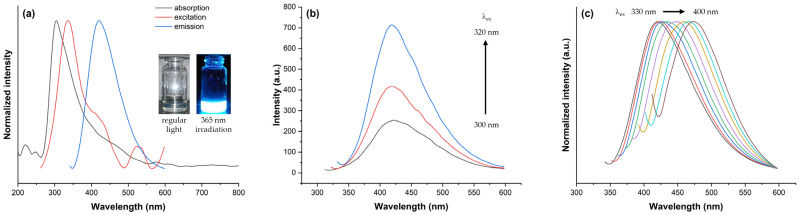
(**a**) The absorption and fluorescence (excitation and emission) spectra of FSCNPs. The inset shows the images when FSCNP is irradiated with regular light (left) and 365 nm light source (left). (**b**) Excitation-independent emission is observed at excitation wavelengths of 300–320 nm. (**c**) Excitation-dependent red-shift emission is observed when the excitation wavelength is varied from 330 to 400 nm.

The optical properties of FSCNPs, including their absorption, excitation, and emission characteristics, were analyzed to better understand their behavior. As is common with doped carbon nanoparticles, FSCNPs display light absorption in the UV–Vis region, ranging from 200 to 600 nm. This is illustrated in Figure 5a, where the maximum absorption wavelength ($\lambda_{max}^{abs}$) is observed at 305 nm. This absorption can be attributed to several factors, detailed as follows: (1) The near ultraviolet region (300–350 nm) is associated with edge or molecular bands resulting from n-π* transitions involving nitrogen- or oxygen-containing structures on the periphery of the carbon network, aligning with previous characterizations. (2) The far ultraviolet region (200–250 nm) corresponds to π-π* transitions of aromatic C=C bonds within the carbon network. Additionally, a broad tail extending into the visible spectrum is commonly observed, likely due to lower-energy surface centers, which are often related to nanoparticle functionalization. This suggests the presence of multiple π→π* (C=C) and n→π* (C=O, C-N, and possibly other) transitions.

The fluorescence properties of FSCNPs were examined through their fluorescence spectra. As illustrated in Figure 5a, the excitation spectra of the FSCNPs display two prominent peaks at 335 nm and 526 nm, along with a shoulder peak at 410 nm. In the emission spectra, the maximum emission wavelength ($\lambda_{max}^{em}$) is observed at 421 nm, with an absolute quantum yield (QY) of 6.3%. These characteristics are attributed to the FSCNPs’ conjugated and non-conjugated structures, domain sizes, and surface states. The FSCNP solution appeared transparent in visible light and was uniformly dispersed with no signs of aggregation or precipitation. Under ultraviolet light (365 nm), the solution showed bright-blue fluorescence (inset Figure 5a). To further evaluate the emission characteristics of FSCNPs, fluorescence scanning was conducted with excitation wavelengths ranging from 300 to 400 nm in 10 nm increments. As illustrated in Figure 5b,c, both excitation-independent and excitation-dependent emissions were detected. Siddique et al. [28] explained that when carbon dots (CDs) are excited with UV light below their threshold excitation wavelength ($\lambda^{ex}$ = 320 nm for FSCNPs), the emission wavelength remains constant. This suggests a single dominant energy level responsible for the emission, likely due to transitions between energy bands within the CD itself. Conceptually, if CDs have a filled “valence band” and an empty “conduction band,” excitation with light of sufficient energy (above the bandgap) causes electrons to jump from the valence band to the conduction band. As these electrons return to their original state, they emit light with energy corresponding to the bandgap. This emission is consistent regardless of the excitation wavelength, provided it is above the bandgap. In contrast, when excitation light surpasses the threshold wavelength, the color of the emitted light varies with the excitation wavelength. This variation suggests the involvement of multiple energy levels in the emission process. Such variability is likely due to transitions involving heteroatom-containing functional groups on the surface and imperfections at the edges of the CDs, which introduce additional energy levels within the bandgap. Excitation with varying wavelengths can promote electrons to these additional levels, and the emitted light will correspond to these specific energy levels, resulting in a wavelength-dependent emission and observed red shift. The insights provided by Siddique et al. [28] are crucial for understanding the multicolor luminescence mechanism of FSCNPs, which will be further explored in the succeeding section.

**Figure 6:**
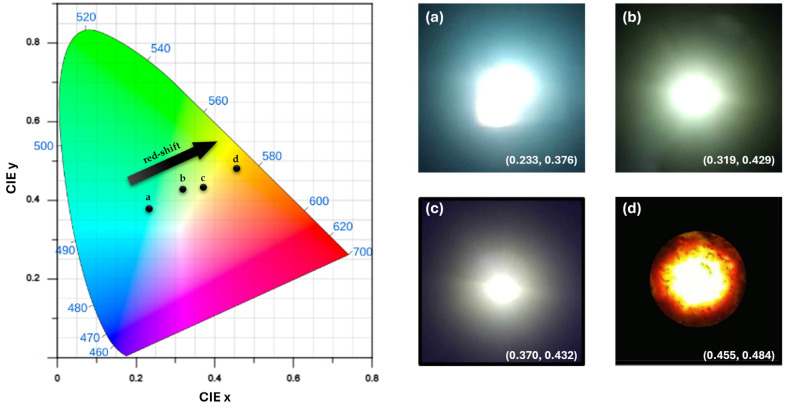
CIE 1931 diagram illustrating the chromaticity of the prepared LED devices. Images (**a**–**d**) show actual photographs of the LED light emissions irradiated at 365 nm, with each image plotted on the CIE diagram along with its corresponding x, y coordinates.

To assess the applicability of FSCNPs in LED devices, various concentrations of FSCNPs were mixed with polyvinyl pyrrolidone (PVP), which served as the solid host matrix. Figure 6 displays the Commission Internationale de l’Éclairage (CIE) 1931 chromaticity diagram of the prepared LED devices, along with actual photos and their x, z coordinates. The figure illustrates the red shift in the emitted light as the concentration of FSCNPs in the film phosphors increased. This change is believed to be due to aggregation-induced emission (AIE); specifically, the concentration-dependent fluorescence effect. Unlike fluorescence quenching, where the intensity decreases, an increase in the concentration of FSCNPs causes a shift toward longer wavelengths. A more detailed explanation of the underlying mechanism will be discussed in the following section.

### 2.4. Luminescence Mechanism of FSCNPs

#### 2.4.1. The Factors, Variables, and Parameters That Contributed to the FSCNPs’ Luminescence

To thoroughly understand the luminescent properties of fish-scale carbon nanoparticles, it is essential to investigate a range of factors and experimental variables. These variables include the characteristics of the precursor molecule, the chosen synthesis method, and the precise control over reaction parameters. The precursor molecule is critical in determining the properties of the synthesized nanoparticles, as factors such as heteroatom content and the presence of conjugated and non-conjugated systems influence the formation of surface traps, defect states, and emissive centers within the carbon dot structure. These structural details directly affect the luminescence behavior of FSCNPs [29,30]. The hydrothermal synthesis method used also plays a vital role, influencing the reaction pathway and subsequently altering the size, shape, and surface chemistry of the resulting carbon dots. These changes significantly impact the luminescence characteristics of the nanoparticles [31,32]. Precise control over reaction time and temperature is crucial as well. These parameters govern the extent of carbonization, functionalization, and the internal rearrangements within the carbon dot structure, directly affecting the energy transitions and emission characteristics of the FSCNPs.

The initial step involves centrifuging the carbonized fish-scale solution obtained from hydrothermal treatment. This process is essential for separating and discarding the insoluble remnants of the charred fish scales, thereby isolating the brown supernatant liquid. The preserved brown liquid is then dried under controlled conditions to maintain its chemical composition and structural integrity, resulting in a fine powder. This powder undergoes characterization, revealing valuable insights into its nature. The FTIR spectral analysis shows the presence of amino, carboxyl, and other hydrophilic groups similar to Type I collagen [10,11,31,33,34], a primary component of the tamban scales. TEM images reveal the nanometer dimensions of the luminescent nanoparticles, distinguishing them from bulk collagen polymer extracts and highlighting their unique electronic properties crucial for multicolor luminescence. The images also show clusters of nanoparticles with a cross-linked network of atoms. It is important to note that FSCNPs may not exhibit the quantum confinement effect. Many researchers have found that carbon dots obtained through bottom-up approaches do not show substantial evidence of quantum confinement but rather exhibit conventional polymeric traits [29,35]. In the case of FSCNPs, the luminescence is likely influenced more by the size of the conjugated and non-conjugated structures formed from the incomplete carbonization of crosslinked polymer clusters rather than by quantum confinement effects [36,37].

Further examination using EDS, XRD, Raman, and UV absorbance spectroscopy confirms the presence of C≡N, C=O, C=N, and C=C groups within the surface and core of the nanoparticles. These functional groups contribute to the non-conjugated and conjugated structures responsible for the luminescence centers, identified as sub-luminophores [35,38,39]. The synthesis conditions, such as a temperature of 180 °C and a duration of 7 h, likely promoted the formation of polymeric clusters during the hydrothermal carbonization of the fish-scale precursor. Incomplete carbonization may have led to the detection of collagen-like hybrid structures instead of highly graphitic forms.

**Figure 7:**
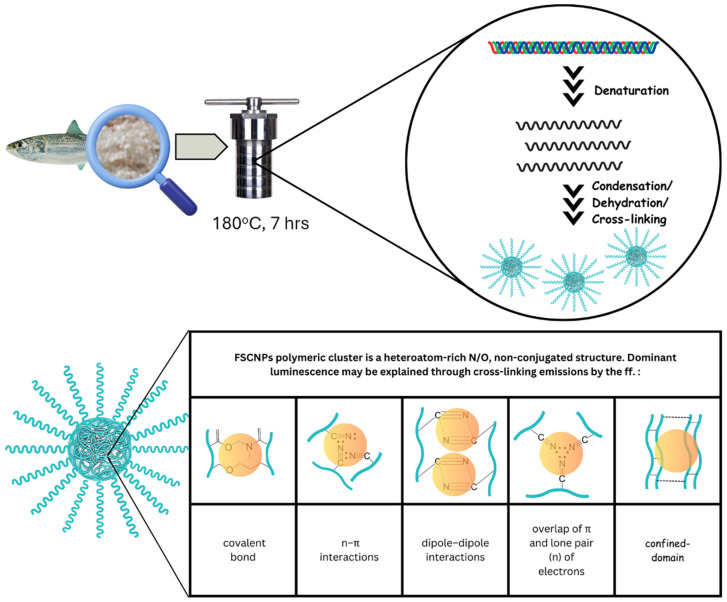
Mechanism of formation and luminescence origin of FSCNPs.

An analysis of the synthesis conditions and material characterization using techniques such as FTIR, TEM, SEM, XRD, Raman spectroscopy, and absorption spectroscopy reveals a clear correlation. The luminescent nanoparticles derived from tamban fish-scale precursors exhibit properties that align closely with those of carbonized polymer dots [38,40]. The proposed luminescence mechanisms of the FSCNPs are summarized in Figure 7. Additionally, while conjugated π-domain emission centers are observed in the absorption spectrum, the emission from non-conjugated structure centers predominates. Surface characterizations demonstrate a variety of functional groups on the FSCNPs, providing them with exceptional versatility, including tunability, solubility, biocompatibility, and stability. This understanding of the surface chemistry, revealed through XPS analysis, sets the stage for developing FSCNPs with multicolor emission, which will be discussed in the next section.

#### 2.4.2. Origin FSCNPs’ Multicolor Luminescence

**Figure 8:**
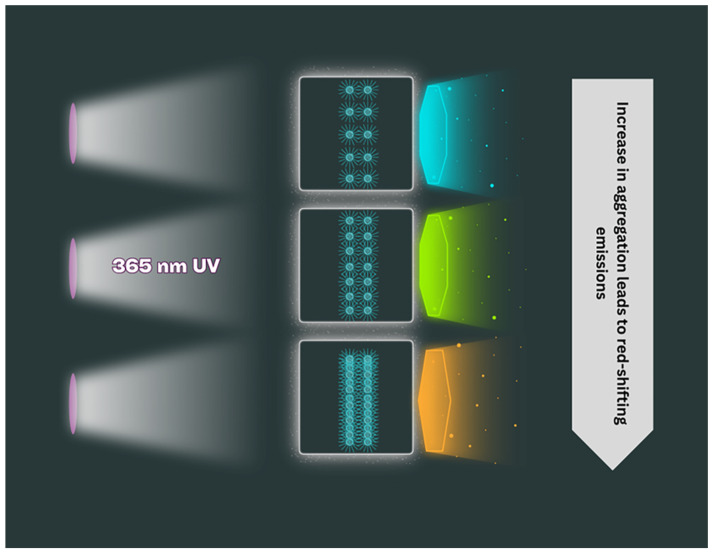
Concentration-dependent fluorescence of FSCNPs: Red-shifted emission and multicolor luminescence from 430 to 635 nm.

A crucial aspect of the photoluminescence of FSCNPs is their retention of abundant functional groups and residual short polymer chains, a result of incomplete carbonization. These residual components provide FSCNPs with numerous highly reactive and customizable sites, enhancing their versatility and modifiability. For example, solid-state FSCNPs exhibit a phenomenon known as aggregation-induced emission, particularly concentration-dependent fluorescence. As the aggregation of FSCNPs increases, the emission shifts from blue to the red-orange region of the spectrum [28,41,42], as presented in Figure 8.

Typically, the luminescence intensity of carbon dots diminishes with higher concentrations. However, FSCNPs show an unusual behavior, namely that their emission wavelength shifts towards longer wavelengths (red shift) as concentration increases. This shift is attributed to the enhanced interactions among aggregated FSCNP particles, which leads to increased non-covalent interactions (NCIs) on their surfaces [43]. Although non-covalent interactions do not directly cause electron delocalization like covalent bonds, they can influence the electronic structure of molecules. This influence includes mechanisms such as hydrogen bonding, π-π stacking interactions, and charge transfer complexes. In particular, π-π stacking, where aromatic rings align and interact, contributes to π conjugation in surface functional groups. When these surface groups are excited by photon absorption, electrons are promoted from lower to higher energy orbitals. In conjugated systems, this excitation often results in electron delocalization over a larger area, leading to increased chances of relaxation through non-radiative processes like internal conversion or intersystem crossing. This mechanism tends to produce red-shifted emissions because the extended conjugated system reduces the energy gap between the excited and ground states, resulting in longer-wavelength emission.

X-ray photoelectron spectroscopy analysis reveals that the sample’s surface chemical states likely facilitate non-covalent interactions. The presence of diverse chemical states, such as carbonyl (C=O) and carbon–oxygen (C-O) bonds in the O1s spectrum, and nitrogen species like pyridinic, pyridinic/amine, and pyrrolic nitrogen in the N1s spectrum, indicates functional groups capable of participating in various non-covalent interactions. Additionally, aromatic carbon structures, as shown by C-C/C=C bonds in the C1s spectrum, further contribute to non-covalent interactions through π-π stacking. This explains why an increased concentration generally leads to a red shift in emission wavelength rather than a blue shift [29,38].

Another important aspect of the multicolor luminescence of FSCNPs, dependent on concentration, is the concept of wavelength threshold [28]. Identifying this threshold is crucial for selecting an appropriate UV light source to excite the FSCNPs. It has been observed that despite differences in structure and composition, all zero-dimensional carbon types exhibit both excitation-independent and excitation-dependent emissions. Therefore, fabricating carbon dot phosphors requires identifying their excitation wavelength through this wavelength threshold to distinguish between excitation-independent and excitation-dependent emission regions.

## 3. Materials and Methods

### 3.1. Materials

Raw tamban fish scales (*Sardinella*, *Clupea* sp.) were collected from Universal Canning Incorporated, located in Zamboanga City, Philippines. Deionized water was used for all experiments. All chemical reagents were of analytical grade and were used without further purification unless otherwise stated.

### 3.2. Sample Pre-Treatment

The raw tamban scales were thoroughly cleaned by washing them in boiling water and constantly stirring them with a mechanical mixer. This process helped to remove any remaining flesh residues, oil, or other impurities. After the first wash, the scales were rinsed a second time in hot distilled water to ensure that all remaining impurities were completely removed. The scales were then dried in an oven set to 60 °C to prevent moisture from affecting the uniformity of particle size. To ensure uniformity, the dried flakes were ground in an analytical mill and sieved through a 60–120 micron mesh sieve.

### 3.3. Sample Preparation

Fish-scale-derived carbon nanoparticles were synthesized from raw tamban fish scales using a standard hydrothermal digestion procedure. Several factors, including reaction temperature, time, pH, and solvent volume, significantly affect FSCNP synthesis and subsequent fluorescence intensity. Deionized water was chosen as the solvent because it is simple and environmentally friendly, and the pH did not need to be adjusted during the experiment. The Box–Behnken design was chosen for optimization because it requires fewer runs compared to full factorial experimental designs and still provides reliable results, which is particularly advantageous for complex or costly experiments. Following the principles of green chemistry, we set lower and upper limits for reaction time (3 and 7 h), temperature (140 and 180 °C), and solvent volume (2.5 mL and 7.5 mL) [17]. These variables, referred to as *X*_1_, *X*_2_ and *X*_3_, respectively, were chosen in accordance with shorter reaction times and lower temperatures. The dependent response, the area under the curve (AUC) of fluorescence intensity, is referred to as *Y′*. To describe the impact of the variables, we utilized a quadratic equation model, as shown in Equation (3).

(3) $$Y^{\prime }=\beta_{0}+\sum_{i=1}^{k}\beta_{i}X_{i}+\sum_{i=1}^{k}\beta_{ii}X_{i}^{2}+\sum_{i=1}^{k}\sum_{j>1}^{k}\beta_{ij}X_{i}X_{j}$$

where the synthesis variables are represented by *X_i_* and *X_j_*, *β*_0_ is the intercept, and *β_i_*, *β_ii_*, and *β_ij_* are the coefficients for linear, quadratic, and interaction effects, respectively [44].

In brief, 0.5 g of fish-scale powder was mixed with deionized water and heated in a 50 mL steel reactor with Teflon lining in a muffle furnace (model: L3/11/B410, Nabatherm, Germany) at a heating rate of 5 °C per minute. After cooling to room temperature, the solution was transferred to a polypropylene centrifuge tube and centrifuged at 8000 rpm for 10 min to separate insoluble residues from the dark brown supernatant liquid. This process was repeated three times to ensure that the residues were completely removed and only the supernatant liquid remained for further use. The resulting liquid was microfiltered (0.22 µm syringe filter) and dried at temperatures up to 80 °C, resulting in a fine powder with a light to dark brown color.

### 3.4. Preparation of FSCNP Phosphors

To evaluate the color conversion efficiency of FSCNPs, we prepared composite thin films of FSCNP-PVP. First, FSCNPs synthesized via a hydrothermal method were dried and finely ground using an agate mortar and pestle to ensure consistent dispersion within the polymer matrix. PVP was chosen as the matrix material for its excellent film-forming properties, transparency, and compatibility with various materials. For the composite film fabrication, 4 g of PVP powder was dissolved in 3 mL of deionized water under constant stirring and gentle heating at 40 °C until complete dissolution. Different amounts of FSCNPs (0.8 g, 1.0 g, 1.33 g, and 2.01 g per 1 mL of PVP solution) were added to the PVP solution and thoroughly mixed to achieve uniformity. This mixture was then poured into a polypropylene mold to control the final film’s shape and dimensions. The mold, containing the FSCNP-PVP mixture, was pressed using a sealing machine (Dyenamo, DN-HM01, Stockholm, Sweden) at 80 °C and 30 PSI, resulting in thin films of uniform thickness (40 µm). These FSCNP-PVP composite films were subsequently exposed to 365 nm UV radiation and characterized through emission measurements using the Avantes AvaSpec-ULS2048CL-EVO spectrometer (Apeldoorn, The Netherlands).

### 3.5. Characterization

#### 3.5.1. Transmission Electron Microscopy 

Given the high solubility of FSCNPs in water, a dilute suspension was prepared by dispersing the nanoparticles in deionized water at a concentration of 0.05 mg/mL. To ensure uniform dispersion and minimize aggregation, the suspension underwent sonication for 15 to 30 min. This process effectively broke up any agglomerates and facilitated the even deposition of the nanoparticles onto the TEM grid. A small volume of the sonicated FSCNP suspension was then carefully drop-cast onto a carbon-coated TEM grid. To further reduce aggregation and ensure a representative sample, only the upper portion of the solution was pipetted for deposition. The prepared TEM grids were subsequently imaged using a JEOL JEM—1400 Plus instrument (Akishima, Tokyo), capturing images at magnifications ranging from 100,000 to 500,000× to resolve the fine details of the FSCNP morphology.

#### 3.5.2. X-ray Photoelectron Spectroscopy 

X-ray photoelectron spectroscopy was conducted to examine the elemental composition and chemical states of the elements within the FSCNPs. Given the non-conductive nature of the FSCNP material, charge compensation techniques were implemented during the XPS measurements to mitigate surface charging effects that could interfere with accurate data collection. The analysis utilized the Thermo Scientific Nexsa G2 surface analyzer, (Waltham, MA, USA), which employs a monochromatic Al Kα X-ray source with a radiation energy of 1486.6 eV. For broad-spectrum survey scans, a pass energy of 200 eV was used to provide an overview of the elemental constituents. In contrast, a higher resolution pass energy of 50 eV was employed to obtain individual core-level photoelectron lines, allowing for a detailed investigation of the chemical states and bonding environments of the elements.

#### 3.5.3. Fourier Transform Infrared Spectroscopy

FTIR spectroscopy was used to identify the functional groups in both the raw material and the synthesized nanoparticles. The samples analyzed included ground and sieved raw fish scales before synthesis, and the fine brown powder of the synthesized fish-scale-derived carbon nanoparticles (FSCNPs). The analysis was performed using a Nicolet iS10 FTIR spectrometer (Thermo-Fisher Scientific, Waltham, MA, USA) with an attenuated total reflection (ATR) accessory featuring a diamond crystal cell. This setup enabled direct sample measurement on the IR detector, eliminating the need for additional preparation like pelletizing with KBr. Each spectrum was constructed from an average of 32 scans per sample and background, spanning a wavenumber range of 4000 to 500 cm^−1^ at a resolution of 4 cm^−1^. The atmospheric interference was minimized by subtracting air background spectra from the sample readings. All measurements were conducted at room temperature.

#### 3.5.4. X-ray Diffraction 

XRD analysis was performed to investigate the crystal structure and phase composition of the FSCNPs. The measurements were carried out using the X-ray Diffraction System—SmartLab by Rigaku (Tokyo, Japan), which utilizes a Cu Kα radiation source. To prepare the FSCNP sample, it was finely ground into powder using an agate mortar and pestle, and subsequently mounted on a glass substrate using a doctor blading technique to create a uniform thin film. Data were collected within a 2θ range of 15° to 80° with a step size of 0.01°. The resulting diffraction patterns were analyzed to determine the phases present in the FSCNPs.

#### 3.5.5. Raman Spectroscopy

Raman spectroscopy was utilized to delve deeper into the structural properties of the FSCNPs. The analysis employed a LabRAM system (Horiba, Palaiseau, France) integrating Raman spectroscopy with Atomic Force Microscopy (AFM). Given the strong fluorescence exhibited by the FSCNP powder, selecting an appropriate laser source for Raman excitation was critical to minimize interference. Ultimately, a 785 nm laser source was chosen for its ability to effectively reduce fluorescence background, enabling the clear acquisition of Raman spectra.

#### 3.5.6. UV–Visible and Fluorescence Spectroscopy

UV–Visible and fluorescence spectroscopy were utilized to investigate the optical properties of the FSCNPs, shedding light on their light absorption and emission characteristics. The UV–Visible absorption spectra of the FSCNPs were recorded using an Avantes AvaSpec-ULS2048CL-EVO spectrometer (Apeldoorn, The Netherlands). Due to the high solubility of FSCNPs in water, a dilute solution was prepared in deionized water, and a small volume was placed in a quartz cuvette for measurement. The absorption spectrum was collected over a wavelength range of 200 to 800 nm. Fluorescence measurements were conducted using a Cary Eclipse fluorescence spectrometer (Agilent Technologies, Victoria, Australia), employing the same FSCNP solution prepared for UV–Visible spectroscopy. A series of excitation wavelengths, ranging from 300 to 400 nm, were used to excite the FSCNPs, and the corresponding emission spectra were recorded over a range of 340 to 600 nm. To further characterize the fluorescence properties, a fluorescence excitation–emission map was generated by scanning both excitation and emission wavelengths within the ranges of 300 to 400 nm and 340 to 600 nm, respectively. For both UV–Visible and fluorescence measurements, the FSCNP solution was prepared by dispersing a small amount of fine brown powder in deionized water at a concentration of 0.3 mg/mL. 

#### 3.5.7. Absolute Quantum Yield Measurement

Absolute quantum yield measurements were conducted to assess the efficiency of FSCNPs in converting absorbed light into emitted light. These measurements were performed using a Hamamatsu Quantaurus-QY Absolute PL Quantum Yield Spectrometer (Hamamatsu, Japan). A solution containing FSCNPs at a concentration of 0.3 mg/mL in deionized water was utilized for this purpose. The instrument utilizes an integrating sphere to capture all emitted photons, ensuring the precise determination of the quantum yield.

## 4. Conclusions

In conclusion, the development of fish-scale-derived carbon nanopolymer dots with multicolor conversion capabilities marks a significant breakthrough in nanotechnology, with promising applications across various industries. This study successfully demonstrated that FSCNPs can be created from fish scales—a readily available and sustainable waste material—resulting in nanoparticles with unique luminescent properties. Through comprehensive characterization techniques, including FTIR, XPS, TEM, EDS, XRD, Raman spectroscopy, and absorption spectroscopy, valuable insights into the structural and chemical properties of FSCNPs were gained. These nanoparticles possess characteristics akin to carbonized polymer dots, such as the presence of amino, carboxyl, and other hydrophilic groups, along with conjugated and non-conjugated structures. The luminescence of FSCNPs is driven by aggregation-induced emission and non-covalent interactions, which contribute to their multicolor emission. The fluorescence of FSCNPs is concentration-dependent, allowing for a bathochromic shift in emission wavelength and enabling a range of emission colors from blue to red-orange when irradiated with a 365 nm light source. This research paves the way for FSCNPs to be used as versatile and customizable materials in optoelectronic devices, bioimaging, sensing, and other fields, advancing sustainable and eco-friendly technologies.

## Data Availability

The original contributions presented in the study are included in the article/supplementary material; further inquiries can be directed to the corresponding author.

## Supplementary Materials

The following supporting information can be downloaded at: https://www.mdpi.com/article/10.3390/ijms252010929/s1.
