# Supplementary material for: Unlocking the Luminescent Potential of Fish-Scale-Derived Carbon Nanoparticles for Multicolor Conversion
Source: Int J Mol Sci. 2024 Oct 11;25(20):10929. doi: 10.3390/ijms252010929 (PMC11507599; doi:10.3390/ijms252010929)
Supplement: Supplementary file 1 [file ijms-25-10929-s001.zip › ijms-3232495-supplementary.pdf]

International Journal of
Molecular Sciences

MDPI

SUPPORTING INFORMATION

Unlocking the Luminescent Potential of Fish-Scale-Derived Carbon Nanoparticles for Multicolor Conversion

Najeeb S. Abdulla II [1,2], Marvin Jose F. Fernandez [2], Bakhytzhan Baptayev [3] and Mannix P. Balanay [3,4,*]

[1] Chemistry Department, Western Mindanao State University, Zamboanga City 7000, Philippines; najeeb.abdulla@wmsu.edu.ph
[2] Department of Chemistry, Mindanao State University-Iligan Institute of Technology, Iligan City 9200, Philippines; marvinjose.fernandez@g.msuiit.edu.ph
[3] National Laboratory Astana, Nazarbayev University, 53 Kabanbay Batyr Ave., Astana 010000, Kazakhstan; bbaptayev@nu.edu.kz
[4] Chemistry Department, Nazarbayev University, 53 Kabanbay Batyr Ave., Astana 01000, Kazakhstan
* Correspondence: mannix.balanay@nu.edu.kz

Table S1. Box–Behnken experimental factors and levels design for optimizing preparation conditions of FSCNPs.

Item	Factor	Level		
		**–1**	**0**	**+1**
X_1	Temperature (°C)	140	160	180
X_2	Time (h)	3	5	7
X_3	DI water volume (mL)	2.5	5	7.5

Table S2. Generated BBD of FSCNPs preparation conditions.

Run order	Code	Temp (X_1, °C)	Time (X_2, h)	DI H_2O volume (X_3, mL)	Intensity (Y, AUC[a])	
					**Measured**	**Fitted[b]**
1	FSCNP-1	160	3	2.5	2872	3679
2	FSCNP-2	160	5	5	10056	9686
3	FSCNP-3	180	5	7.5	12055	15977
4	FSCNP-4	140	5	7.5	4115	3910
**5[c]**	**FSCNP-5**	**180**	**7**	**5**	**85743**	**60605**
6	FSCNP-6	160	7	2.5	11574	11956
7	FSCNP-7	160	7	7.5	10955	10307
8	FSCNP-8	160	5	5	10573	9686
9	FSCNP-9	180	3	5	9839	9340
10	FSCNP-10	180	5	2.5	29152	30708
11	FSCNP-11	140	7	5	4911	5755
12	FSCNP-12	140	5	2.5	3811	3514
13	FSCNP-13	160	3	7.5	2590	2471
14	FSCNP-14	160	5	5	10020	9686
15	FSCNP-15	140	3	5	2684	2754

[a] AUC – Area under the curve measured within the wavelength range of 360-570 nm.
[b] Fitted/Predicted intensity from the original response.
[c] FSCNP-5 produced response the highest AUC among the FSCNPs 1-15, for the combination of variables (X_1, X_3, X_3). This condition provided the basis for utilizing the parameters in the preparation of FSCNPs.

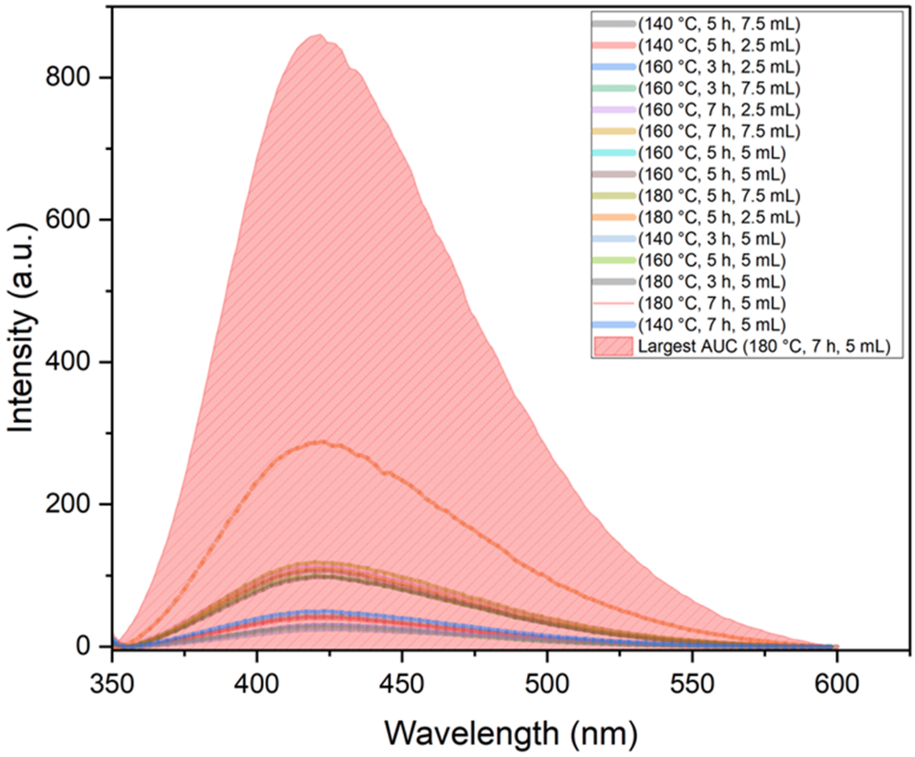

Figure S1. Graphical illustration of the area under the curve measurement based on Table S2. The figure emphasizes the largest AUC from the set of combination (X_1, X_3, X_3).

Table S3. Terms for the response surface model regression equation.

Source	Contribution	Adj. SS	*F*-value	*p*-value
Model	97.20%	33.5347	118.21	0.000
**Blocks**	**0.28%**	**0.0978**	**3.45**	**0.072**
Linear	86.43%	3.4678	40.75	0.000
Sample amount	1.30%	1.1556	40.73	0.000
Temperature	55.58%	0.7038	24.81	0.000
Time	29.55%	1.6084	56.70	0.000
Square	6.30%	2.1748	25.55	0.000
Sample amount*Sample amount	2.80%	0.9539	33.63	0.000
Temperature*Temperature	1.92%	0.5694	20.07	0.000
Time*Time	1.58%	0.5458	19.24	0.000
2-Way Interaction	4.18%	1.4433	16.96	0.000
Sample amount*Temperature	1.26%	0.4335	15.28	0.000
**Sample amount*Time**	**0.14%**	**0.0468**	**1.65**	**0.208**
Temperature*Time	2.79%	0.9630	33.94	0.000
Error	2.80%	0.9646		
Lack-of-Fit	2.36%	0.8139	6.84	0.000
Pure Error	0.44%	0.1507		
Total	100.00%			

Note: The highlighted terms are not significant (p-value > 0.05). Thus, these terms may not significantly affect the response variable. However, the model explains a significant portion of the variance based on the predicted/fitted intensity as indicated in Table S2 (fitted intensity column)

Row a: Effect of varying DI water amount

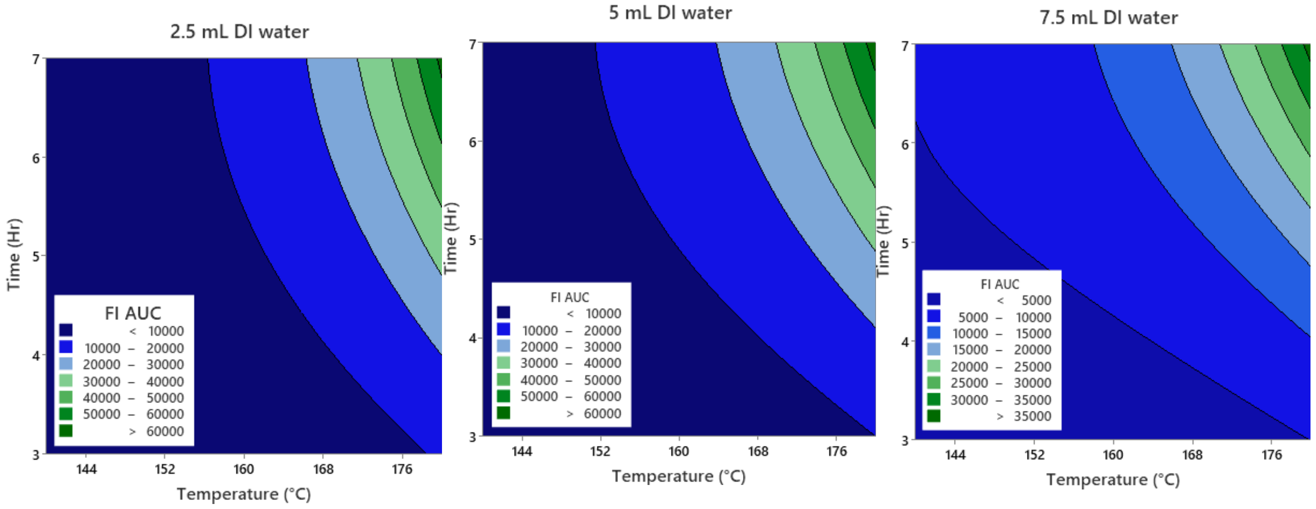

Row b: Effect of varying the heating duration (time):

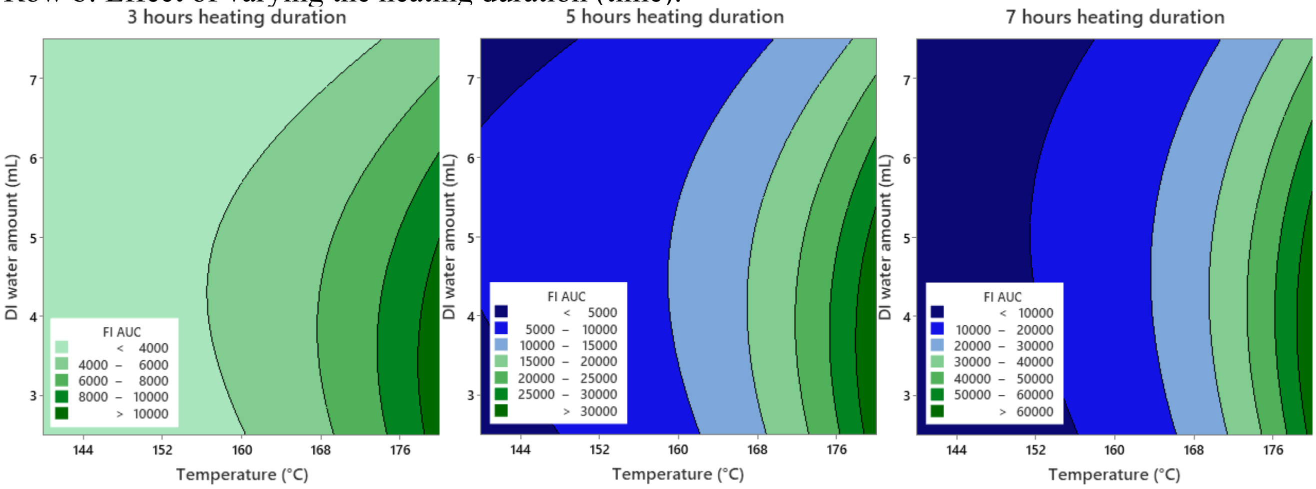

Row c: Effect of varying heating temperature:

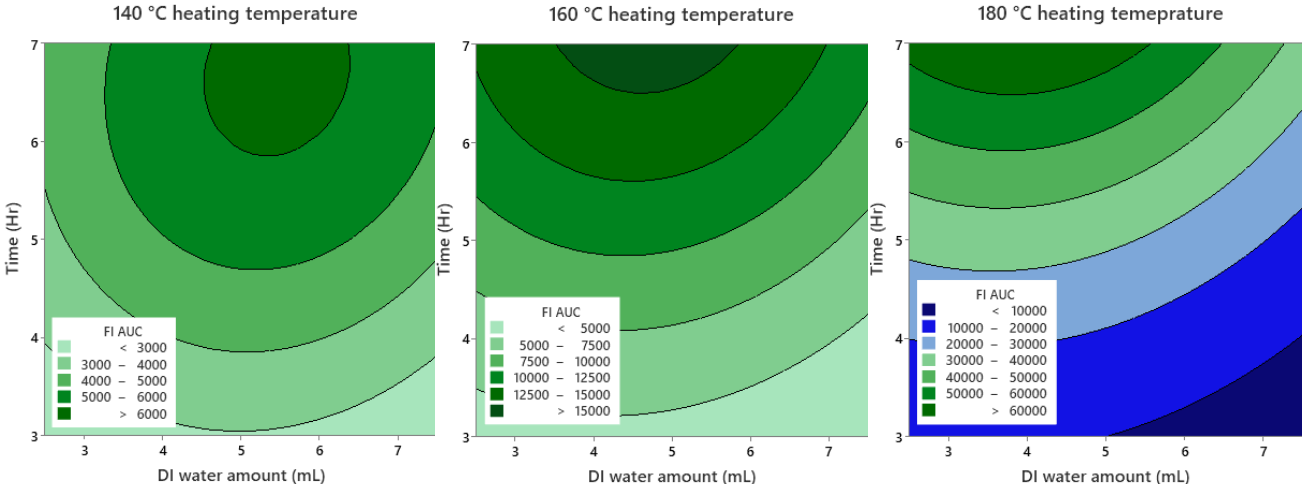

Figure S2. Contour plots of the effects of: (Row a) DI water amount, (Row b) time, and (Row c) Temperature on fluorescence intensity (FI AUC) of FSCNPs.

Table S4. Statistical calculation solutions using the RSM BBD algorithm to find the optimized parameters.

Solution	DI water (mL)	Temperature (oC)	Time (Hr)	FI AUC Fit	Composite Desirability
1	3.87355	180.000	7.00000	67578.4	0.782007
**2**	**4.92119**	**180.000**	**7.00000**	**64190.1**	**0.741343**
3	7.49952	179.999	7.00000	36459.9	0.408546
4	3.86000	180.000	3.00031	11027.3	0.103324
5	7.49725	150.349	6.98706	7088.8	0.056056
6	3.86000	140.000	6.52840	5461.9	0.036531
7	3.86129	140.005	6.99993	5398.1	0.035765
8	7.49822	140.004	6.99999	5065.1	0.031769
9	7.49404	179.992	3.00159	4974.2	0.030679

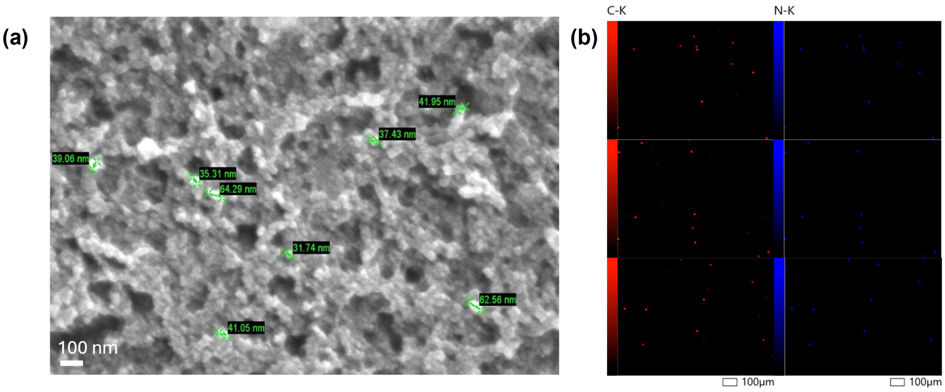

(c)

Element	Mass%	Atom%
C	6.35±0.27	15.14±0.65
N	6.76±0.42	13.81±0.85
O	30.63±1.77	54.81±3.16
Si	3.42±0.29	3.49±0.29
Sn	52.84±1.36	12.75±0.33

Figure S3. (a) SEM image of the FSCNPs, showing luminescent clustered nanoparticles with sizes ranging from 31-64 nm. (b) Elemental mapping of C-N, showing C-N rich regions. (c) Elemental composition with approximately 1C:1N ratio.
